# Regulatory T Cells and Acute Lung Injury: Cytokines, Uncontrolled Inflammation, and Therapeutic Implications

**DOI:** 10.3389/fimmu.2018.01545

**Published:** 2018-07-09

**Authors:** Shihui Lin, Hua Wu, Chuanjiang Wang, Zhibo Xiao, Fang Xu

**Affiliations:** ^1^Department of Emergency and Critical Care Medicine, The First Affiliated Hospital of Chongqing Medical University, Chongqing, China; ^2^Center for Cognitive and Neurobiological Imaging, Stanford University, Stanford, CA, United States; ^3^Department of Radiology, The First Affiliated Hospital of Chongqing Medical University, Chongqing, China

**Keywords:** regulatory T cells, acute lung injury, acute respiratory distress syndrome, uncontrolled inflammation, cytokines

## Abstract

Acute respiratory distress syndrome/acute lung injury (ALI) was described in 1967. The uncontrolled inflammation is a central issue of the syndrome. The regulatory T cells (Tregs), formerly known as suppressor T cells, are a subpopulation of T cells. Tregs indirectly limits immune inflammation-inflicted tissue damage by employing multiple mechanisms and creating the appropriate immune environment for successful tissue repair. And it plays a central role in the resolution of ALI. Accordingly, for this review, we will focus on Treg populations which are critical for inflammatory immunity of ALI, and the effect of interaction between Treg subsets and cytokines on ALI. And then explore the possibility of cytokines as beneficial factors in inflammation resolution of ALI.

## Introduction

After the initial description of acute respiratory distress syndrome (ARDS) (1967), great progress has been made in the study of the pathophysiology and pathogenesis of acute lung injury (ALI) (1–5), and much has been learned about the pathogenesis of lung injury in ARDS. Uncontrolled inflammation is a central issue in ALI, and understanding how this is regulated is important for developing new treatment strategies for limiting excessive inflammation.

Throughout human life, T cell system adjusts to the transfer of resources and needs, resulting in a fundamentally reorganized immune system in each individual (6). In ALI, central to these issues is the large variety of T cells implicated in the pathobiology, pathophysiology, and integration of innate and adaptive immunity leading to uncontrolled pulmonary inflammation. Regulatory T cells (Tregs) are a special type of T cell. It could restrict inflammation-induced tissue damage *via* multiple mechanisms indirectly, such as their anti-inflammatory and antiapoptotic abilities (7), to create an appropriate immune microenvironment for the repairing and regeneration of tissue (8). And it is possible that a kernel mechanism of inhibition may be commonly used by Tregs at any location (e.g., spleen, blood, and lung) or in any inflammatory environment or phase (9, 10).

In order to understand the intricate and dynamic interactions between functionally activated Tregs and the pathophysiology of ALI, it is necessary to analyze the function of specialized T cells in uncontrolled inflammation, the ultimate goal being to identify new therapeutic approaches. In this review, we confine our discussion to Treg populations that play a critical role in the development of uncontrolled inflammation in ALI.

## Uncontrolled Inflammation: A Core Issue in ALI

At present, it is generally accepted that ALI is characterized by rapid alveolar injury, inflammation, cytokine induction, and neutrophil accumulation, with an emphasis on the mechanisms of injury to the lung endothelium and the alveolar epithelium. ARDS represents a stereotypic response to various etiologies. It progresses through different phases, starting with alveolar capillary damage, progressing, to lung resolution, and culminating in a fibroproliferative phase. A hallmark of the damage seen in ARDS is that it is not uniform. A major challenge faced in a clustering approach is the heterogeneity of ALI, not only between patients but also in the same patient from a different cause or at different stages of the disease. All of these complex factors make it extremely difficult to improve the understanding about pathogenesis and treatment in ARDS.

The core mechanisms of ALI may involve trauma, tissue damage, infection, shock (except for cardiogenic shock), or conserved microbial or molecular patterns that trigger receptors on innate immune cells. As a result, series of pro-inflammatory and anti-inflammatory cytokines (or chemokine) are produced.

Recent meta-analyses about a network clinical trials of ARDS, including the measurements of blood biomarkers and clinical indicators, indicate that approximately 35% of patients with ARDS have high inflammatory endocrine, which related to higher mortality (11). Cytokines are proteins with pro-inflammatory or anti-inflammatory effects. Its biological effects need to be completed by autocrine and paracrine manner. Several studies have also reported a number of cytokines—such as TNF-α (12, 13), IL-1β (14), IL-6 (15), IL-17 (16, 17), and IL-33 (18, 19)—were increased in acute stage of ARDS/ALI. Based on these findings, which point to the inflammatory complexity of this disease, ALI must be seen as a syndrome with many manifestations both systemic and pulmonary. By focusing on and exploring the uncontrolled inflammation of ARDS, we hope to come closer to defining its causes and development.

## Treg: T Cells with Anti-Inflammatory Functions

Response to exogenous or endogenous insults, the host brings a range of changes characterized by transform in immune function and the produce of mediators called cytokines. A large number of studies have found that these immunoregulatory mechanisms are beneficial in that they prevent the worsening of injury and promote its repair (20–22). Data also show that inflammatory cytokines contribute to the development and progression of many immunologic and inflammatory diseases (23, 24), suggesting that therapeutic actions may be promoted by regulating these mediators. But the immune system—divided into innate and adaptive branches—comprises many different cell types that vary by both function and topographic location. T and B lymphocytes constitute the major cellular components of the adaptive immune response (25). More than any other cellular system, cell-mediated immune responses are largely controlled by T cells. Environmental factors have frequently been associated with providing the trigger that enables or enhances the development of T-cell immunity in sensitive individuals (26). Many additional aspects of T-cell function can be altered by the local cytokine or chemokine environment. Therefore, by exploring the pathogenesis of uncontrolled inflammation in ALI, we may be able to clarify the functioning of T-cell immunology.

Several Th subsets within the T-cell immune system are now well defined, including Th1, Th2, Tregs, follicular helper T cells, Th17, Th22, and Th25 (26–28). A central question in T-cell immunology is how this group of CD4 T cells can coordinate such diverse immunologic processes involving so many different cell types. A central question in T-cell immunology is how this group of CD4 T cells can coordinate such diverse immunologic processes involving so many different cell types. Tregs are a subtype of CD4^+^ T cells known to be significant for immune homeostasis and maintaining self-tolerance; they were termed suppressor cells originally (29). On the basis of the relative differentiation state and the tissues where Tregs are generated primarily, they can be separated into several subsets, including two major subgroups. One subgroup is a distinct lineage (tTregs) from thymus, the other originate from the peripheral conversion of naive CD4^+^CD25^−^ (Tconvs) into Tregs (pTregs) induced by FoxP3 (30). The interaction of Naive or resting Tregs are lower than the threshold with full activation, but the changes of effector Tregs in surface markers and enhanced suppression. Memory Tregs reacted against antigen and had survivability for quite a time indeed in the absence of antigen apparently. In the suspicion, memory Tregs alleviate tissue damage during the deepen reactions of pro-inflammatory memory cells (31, 32). In the research about ALI, it is an unknown area. However, from the aspects of lung development, pulmonary fibrosis, lung injury and repair, etc., which controlled by inflammation, the characteristics of Treg subtypes should play an important role in ALI and need to be confirmed.

Human Tregs were first characterized as CD4^+^CD25^+^ T cells in 2001 (33, 34); subsequent studies have confirmed transcription factor FoxP3 as a specific regulatory marker of human Tregs (35), which are believed to be important for maintaining immune homeostasis. Numerous publications have reported that the reduced generation or deficient function of Tregs is associated with greater disease severity and activity, as noted in patients with various inflammatory diseases (36). Current studies have also shown that Tregs can limit effector T-cell function (37, 38), which is known to infection control or mediating inflammatory injury (39). The most critical population of Tregs, which expresses Foxp3, can limit the activation, proliferation, and effector roles in series of immune cells (40–42). CD4^+^CD25^+^Foxp3^+^ Tregs are a representative cell type. And it is one of the powerful immunomodulators for adaptive immune system, functionally differentiated to be able to control Th1- or Th2-type immune responses by regulating the expression of Th1- or Th2-related transcription factors specifically as well as the activation of monocytes/macrophages (43, 44). However, although each subset of Tregs was functionally suppressive, they displayed unique inflammatory response patterns about inflammatory factor (pro- or anti-) formation, expressed lineage-specifying transcription factors differentially, after comprehensive functional analysis about various Tregs subsets (27). Studies showed that there is a difference between CD4^+^CD45RO^+^CD25^hi^CD127^lo^ Tregs and CD4^+^CD45RO^+^CD25^−^ Th cells in chemokine (C-C motif) receptor 6 (CCR6), CXCR3, CCR4, and CCR10 from peripheral blood. It could not find the CCR4^−^/CCR4^−^CCR10^−^ phenotypic in the above type of Tregs. Due to their phenotypic similarity with different effector T cell subsets, we considered these populations as Th1-, Th2-, Th17-, and Th22-like Tregs. As described in Th cells, these acquisition of homing receptor phenotypes probably happens after the activation of periphery Tregs (27).

Although natural (n)Tregs are usually stable and long-lived, Tregs may demonstrate instability under pathogenic or inflammatory circumstances (45), and the stability and plasticity of Foxp3 has been under debate. Tregs instability is marked by the loss of Foxp3 expression and suppressive capacity as well as acquisition of features reminiscent of exFoxp3 cells (effector T cells by CD25^lo^Foxp3^+^CD4^+^ T cells lose Foxp3 expression) in response to environmental cues (45, 46). Studies showed that exFoxp3 Th17 cells, characterized by the expression of Sox4, chemokine (C-C motif) ligand 20, CCR6, IL-23 receptor, and receptor activator of NF-κB ligand, were more potent osteoclastogenic T cells than naïve CD4^+^ T cell-derived Th17 cells in autoimmune arthritis (RA) (46). On other hand, the balance between IL-2 and IL-6 should regulate the development of Treg and Th17 cells from naive CD4^+^ T cells (47), and the fate of plastic Foxp3^+^ T cells may be determined by the cytokine balance too (48). Moreover, Th17 cells were shown to transdifferentiate into another Tregs subset, IL-10^+^ T regulatory type 1 cells during the resolving of inflammation (49). An additional source of Tregs includes Th17 cell transdifferentiation into ex-Th17 IL-17A^neg^Foxp3^+^ cells (50). It can be seen that the instability of Tregs in an inflammatory state plays a vital function in the occurrence of diseases. In many autoimmune inflammatory conditions, Tregs plays an anti-inflammatory role, which when it fails, also leads to the development of inflammatory sickness. In several lung diseases, Tregs are in capacity of anti-inflammatory by the contact dependence inhibition or release of cytokines (IL-10, TGF-β1, and INF-γ) mainly (29, 51–53). The expression of the required receptors making Tregs susceptible to these modulating cytokines is initiated only after activation and possibly also some lineage commitment. In addition and more importantly, the number and functional status of Tregs that could produce different cytokines (IL-10 and IFN-γ) or small-molecule proteins (perforin and granzyme) were not the same in different tissues or microenvironments (51, 54, 55). In other words, Tregs can bring into play their functions through a variety of inhibitory mechanisms; however, exactly how Tregs employ these mechanisms in ALI remains unclear.

## Tregs in ALI: Metronome of “Inflammatory Factor Storm” Regulation

**Figure 1:**
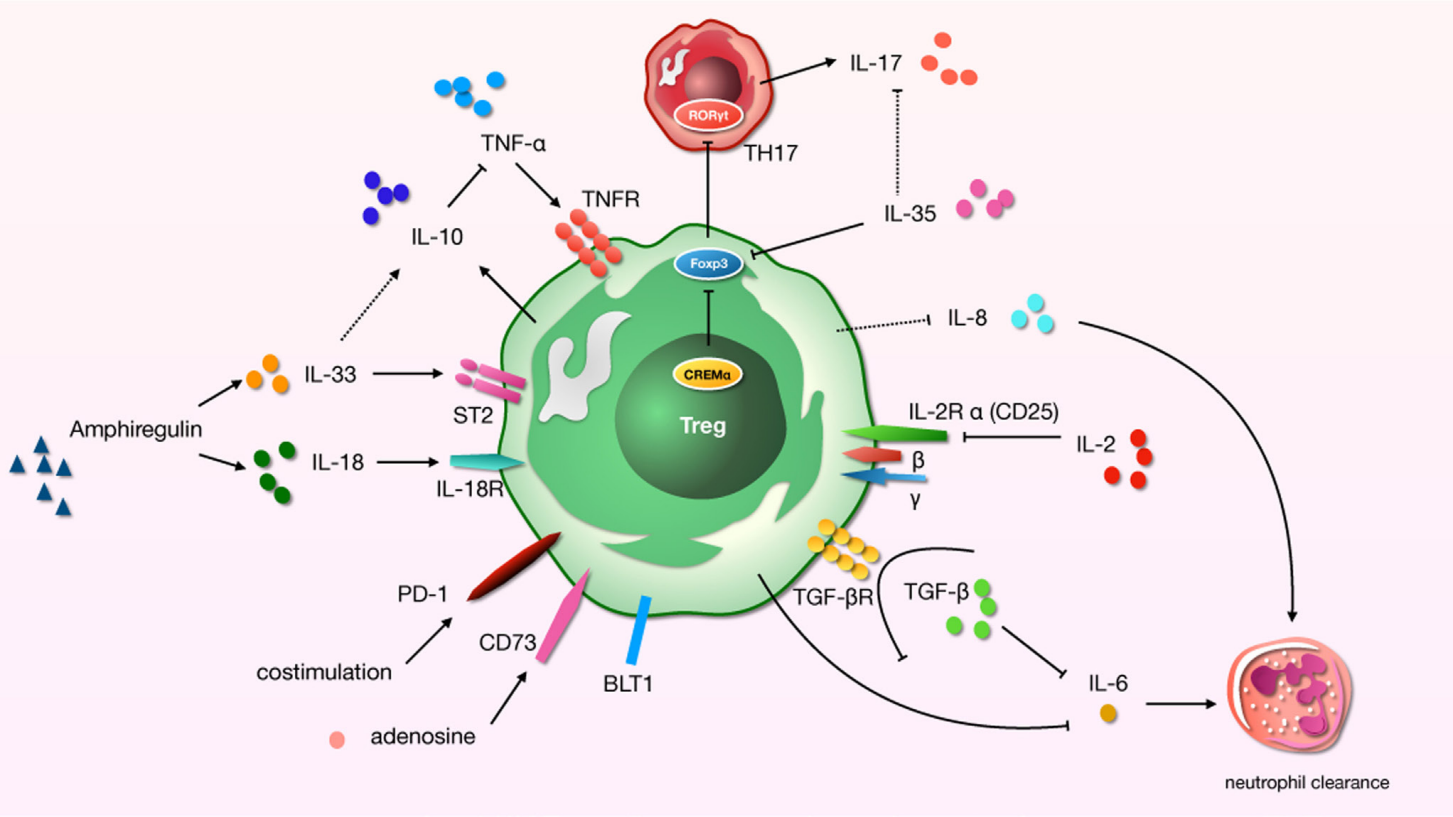
A schematic about regulatory T cells (Tregs)-related cytokines and immunosuppressive molecules in acute lung injury (ALI) process. “→”: promote; “⊥”: inhibit; “⋯”: may play a role.

An infaust outcome of ARDS is related to the initial excessive pulmonary inflammation, which continues over time (56). Many modulators of inflammation were found to be increased in patients with high risk of ARDS who later died. And cytokines play a significant role in the development of lung region immunity. For distinct effector cytokines, Tregs should act as a “cytokine sink.” And inflammation-specific Tregs work out an environment-specific inhibitory activity by the function. Studies have demonstrated a core role for Tregs in the alleviating or treating of ALI/ARDS in that they orchestrate a complex series of therapeutic events (20, 57). We now know that even in the pathophysiology of indirect ALI—which we used to described as a pro-inflammatory pathology mediated by cells of the innate immune system—cells of the adaptive immune response play a major role (58) (Figure 1).

## TNF-α and IL-10

The role of TNF-α as a major test indicator chosen for almost all clinical or basic research on the uncontrolled inflammation of ARDS is both important and obvious. Although there was a little difference in the increase of TNF-α in lipopolysaccharides (LPS) and oleic acid (OA)-induced ALI models, the overall increase was significant [except for the arterial or mixed venous blood samples of OA in pig models (59)]. Research has shown that cure with TNF-α-specific antibody restored Tregs function in rheumatoid arthritis, which was related to decreasing the expression of protein phosphatase 1 and increasing the phosphorylation of Foxp3 in Tregs (60). Current research on ARDS has confirmed that transplantation of human umbilical cord mesenchymal stem cells improves ALI by increasing the alveolar Tregs and balancing pro- and anti-inflammatory factors (including TNF-α) in ALI model (61).

IL-10 is an anti-inflammatory cytokine. Tregs can produce IL-10 to suppress hypernomic immune responses and thus to protect the host (62). This may have an important protective role in ALI, as by ameliorating the lung tissue injury by restraining production of TNF-α and neutrophil activity (63), IL-10 is produced during lung injury and significantly contributes to rapid early immunopathogenesis. During indirect ALI, the regulatory mechanisms of lung activated are mediated by the specific subgroup of CD4^+^CD25^+^Foxp3^+^ Tregs that are central to the domination of neutrophil recruitment by increasing the production of IL-10 (58). In transfusion-related ALI, CD11c^+^ dendritic cells and Tregs were the key effectors in regulating IL-10 (64). Interestingly, analyses in mice have shown that IL-10 production by Tregs was not required to control systemic autoimmunity, but it was necessary for keeping immunologic responses at the environmental/lung interface (51). Obviously, the IL-10 expression is regulated by Tregs in the progress of ALI, and sometimes the effect of IL-10 may inhibit the TNF-α production.

## TGF-β, IL-6, and IL-2

The development of Tregs requires TGF-β, while IL-6 and IL-2 are pivotal regulators obstructing the polarization of Tregs from naive T cells (65, 66). In the research using a mouse model of ALI, the presence of Tregs led to the increased local expression of TGF-β (20). TGF-β participates in many of the pathophysiologic processes of ARDS by stimulating the proliferation of fibroblasts, leading to the development of pulmonary fibrosis (67) and promoting the internalization of the αβγ epithelial sodium channel complex from the alveolar epithelial cell surface. This process leads to persistence of pulmonary edema (68, 69). As we know, lymphocytes can contribute to the resolution of inflammation through the clearance of alveolar neutrophils, which is the mechanism of pulmonary inflammation in ALI and ARDS. Research has confirmed that the neutrophil clearance rate can accelerated by Tregs (4, 20). IL-6 seems to be a reliable biomarker of severity of illness in critical patients with high risk for ARDS, and it was found increased in the bronchoalveolar lavage fluid (BALF) and plasma in several ALI models (70, 71). Early studies showed that IL-6 strengthens the cytotoxic potential of polymorphonuclear leukocytes *via* selective enhancement of elastase release (72). TGF-β functions as an inflammatory pathway of uncontrolled inflammation *via* p38MAPK, SMAD3 (73), or IL-6 to promote neutrophil clearance (74). Thus, Tregs inhibit the uncontrolled inflammation of ARDS through the secretion of TGF-β, which affects IL-6 and then clears the neutrophils. But the debate continues regarding whether it acts as a counterregulatory mediator or pro-inflammatory principally. Follow-up studies have confirmed that IL-6 markedly diminishes lung injury in a hyperoxic model associated with the induction of Bcl-2 and TIMP-1 (75). According to the research of D’Alessio et al., Tregs orchestrated critical events to recovery *via* adoptive transfer after LPS-induced (intratracheal) ALI (20, 76). Interestingly, the absence of Tregs might contribute to the increased expression of the IL-6 gene persistently (76). Thus, the mechanism of Tregs reliance on IL-6 seems to differ in different types of ALI. On the other hand, low doses of IL-2 are the most successful strategy to “boost” Tregs *in vivo* so far (77). IL-2 is necessary for the development and maintenance of Foxp3^+^CD4^+^ Tregs, which prevent the development of autoimmune disease (78, 79). The levels of IL-2 and Treg-related cytokines in BALF and serum gradually decreased in ALI over time (80). Animal studies have shown that in chorioamnionitis, systemic treatment with IL-2 expanded Tregs preferentially by increasing the ratio of FoxP3^+^/CD3^+^ in fetal lungs, thus improving lung function and modulating pulmonary inflammation (81). However, there is little evidence of ARDS/ALI induced by other causes.

## IL-8

IL-8, a potent neutrophil attractant and activator, was found to be increased in the BALF of patients at risk who ultimately developed ARDS (82). A multiple logistic regression model incorporating oxygenation index, IL-8, and TNF-R2 was superior in predicting the composite outcome of mortality or severe morbidity (83). IL-8 plays a significant role in ALI *via* the formation of anti-IL-8 autoantibody, and IL-8 complexes and of those complexes’ interactions with FcγRIIa receptors, leading to the development of ALI by effecting neutrophil apoptosis (84, 85). Limited research has found that Tregs exert an inhibitive effect on innate immune responses and neutrophil infiltration mediated by IL-8 (86). There is also evidence that decreased IL-8 in the BALF and improvement of lung injuries are accompanied by increased CD4^+^Foxp3^+^ Tregs number and Foxp3 transcription level of the lungs (87). But this evidence is of limited significance for the regulation of Tregs in ALI.

## IL-33 and IL-18

The role of IL-33 is not limited to Th2 response. By contrast, IL-33 is a potent (83) activator of group 2 innate lymphoid cells, Th1 cells, Tregs, and CD8^+^ T cells (88), and it is an immunostimulatory factor used to induce Treg expansion (89). It is released in the stage of tissue injury in sepsis and activates type 2 innate lymphoid cells that encourage the polarization of M2 macrophages. In this way, IL-10 enhances the amplification of Treg population (90). In ARDS, IL-33 level of serum were higher in patients with pulmonary factors; then pulmonary inflammation and injury were reduced by the treatment of IL-33 neutralizing antibody (19). In the lung, CD4^+^Foxp3^+^ Tregs expressed ST2, the IL-33 receptor. If exposed to IL-33, Tregs can upregulated the expression of GATA3 and ST2, then produced type 2 cytokines (91). In other studies, selective Tregs deficiency in amphiregulin cause the severe ALI and hypoxias during influenza virus infection; this mobilized Tregs in response to the IL-33 or IL-18 but not by T cell receptors signaling (7, 92). Early-stage serum IL-18 levels are among the signs reflecting the prognosis after 60 or more days in patients with ARDS (93). The pro-inflammatory role of IL-18 in ALI is related to the activation of NLRP3 through the activation of caspase-1 (94, 95) and the upregulation of IL-18-mediated neutrophil infiltration (96).

## Th17, IL-17, and IL-35

A balance between Th17 and Tregs may be essential for maintaining immune homeostasis and has long been thought to be an important factors in the development/prevention of autoimmune and inflammatory diseases (97). The severity of lung injury was associated with imbalanced T-cell subsets, which were related to the combined effect of increased pro-inflammatory (Th1) cells and decreased anti-inflammatory cells (Foxp^3+^ Tregs) (98). Th17/Treg imbalance favoring a Th17 shift indicates a potential therapeutic target to reduce lung injury and a novel risk indicator for early ARDS patients (80, 99). Meanwhile, IL-17-producing cells (Th17) have a pro-inflammatory effects. Studies have shown that elevated level of IL-17A in alveolar and circulating related to the increased percentages of alveolar neutrophils, greater alveolar permeability, and reduced organ dysfunction in ARDS (97, 100). We must pay attention to cyclic AMP-responsive element modulator-α (CREMα), overexpression of CREMα in T cells should change the inflammatory microenvironment. Levels of CREMα in T cells could determine the outcome of ALI, and upgraded CREMα contributes to increased IL-17 expression and decreased Foxp3, IL-2, and numbers of Tregs (101). Non-ALI research shows that IL-17-producing biTregs coincide with the progress of immuno-inflammation and tissue injury, and the specific elimination of RORγt activation in endogenous biTregs results in the improvement of pulmonary vasculitic injury (102). The evidence we were able to find about ALI is limited to pentoxifyllinum-induced increased cAMP in ARDS, which may restore the balance of Treg/Th17 *via* the transcriptional regulation of RORγt and Foxp3 partly through STAT3 signal (103).

IL-35 is a relatively newly identified cytokine obligatory for the regulatory and inhibit role of Tregs; it plays a vital function in prevention and cure in autoimmune diseases. One study shows that the degree of lung injury mitigation was accompanied by increased CD4^+^Foxp3^+^ Tregs number as well as the Foxp3 transcription level of lung and IL-35 level from the BALF in rats (87). But there is no direct research of the role and mechanism about Tregs and IL-35 in ALI. However, a study of pulmonary inflammatory disease (allergic airways disease) confirmed that IL-35 production by inducible costimulator-positive Tregs can suppress IL-17 production and thereby reverse the inflammation (104). Thus, the effect of IL-35 may be exerted by altering the expression of IL-17.

## Other Immunosuppressive Molecules

Other immunosuppressive molecules also participate in the progress of ALI. In mice and patients with ALI, the alveolar recruitment of Tregs specifically mediated by the leukotriene B4–BLT1 pathway contributed to the resolution of lung inflammation (20, 105), particularly in the resolution of ALI fibroproliferation (21). And Tregs can modify the ALI-induced innate immune response directly, a process mediated in part by PD-1 (106). Furthermore, Ecto-5′-nucleotidase eNT (CD73)^+^ Tregs have been found to contribute to adenosine-mediated resolution of ALI (107).

## Conclusion and Future Perspectives

**Figure 2:**
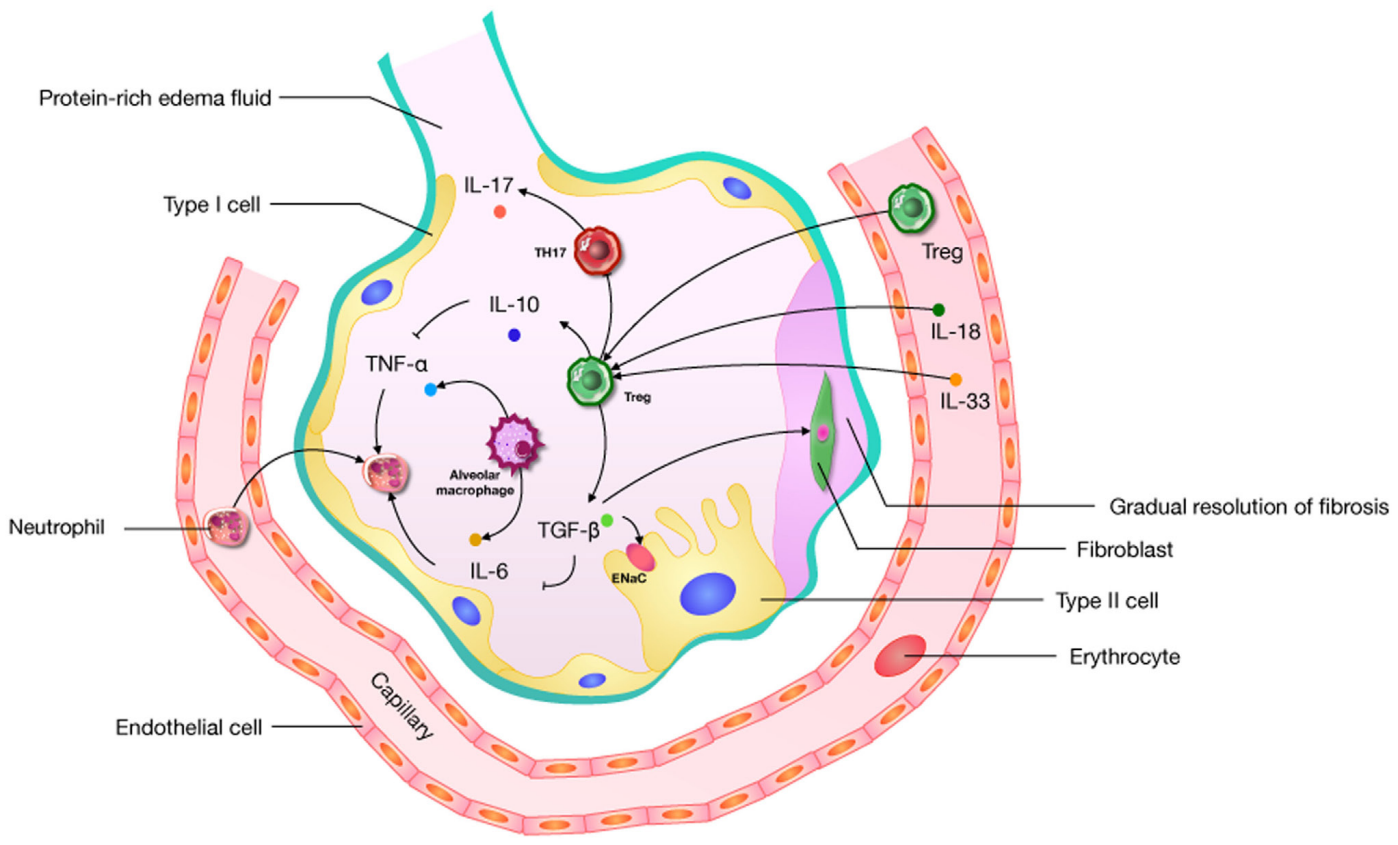
A pathophysiological schematic about regulatory T cells (Tregs) improving the acute lung injury (ALI) process through the influence of cytokines (including TGF-β, IL-6, IL-10, IL-17, IL-18, and IL-33) which has been confirmed. ENaC: epithelial sodium channel; “→”: promote; “⊥”: inhibit.

The diverse etiologies of ARDS limit the study of its mechanisms and therapies. Given the heterogeneity of ARDS, we may well be forced to focus on just one of its features, namely uncontrolled inflammation. Cytokines play a crucial role in creating an immunogenic microenvironment and therefore are key players in the promotion or inhibition ALI. Complex networks of cytokines and chemokines regulate the progression of ARDS. An activated immune system and immune cells (CD4, CD8, Th1, Th2, Th17, Tregs, and others) act as a significant role in this network. The role of Tregs is to prevent the development and extension of inflammatory diseases (36), and they can prevent the development of immune pathology and inflammation in the environmental/pulmonary interface involved in ALI. Based on the effect of uncontrolled inflammation in the promotion of ALI, it is possible that the disease’s microenvironment may offer useful cues regarding Tregs, but this remains unclear. Some research suggests that Tregs can improve the ALI process through the influence of TGF-β (68, 69, 73, 74), IL-6 (20, 76), IL-10 (51, 58, 64), IL-17 (101), IL-18 (7, 92), and IL-33 (91) (Figure 2). What needs to be raised is that IL-17, generated by Th17 cells preferentially, has an abundant research about ARDS (108). The Th17/Treg balance toward Th17 cells might improve the aggregation of inflammatory mediators, and forming an amplificatory inflammatory loop to promote uncontrolled inflammation in patients with ARDS (99). In an IL-6-rich inflammatory microenvironment, Th17 enhanced while Tregs are suppressed (109). So the role of IL-6 in shifting Th17/Treg axis in ALI is worth noticing. And IL-6 as a target for the treatment about Tregs should be considered.

There has also been some indirect evidence that Tregs have an impact on TNF-α (60, 61), IL-2 (81, 101), and IL-8 (87). In addition, some immunosuppressive molecules [BLT1 (20, 101), PD-1 (106), and CD73 (107)] have also been shown to play an important role in Treg immune function in ALI. Hence, the manipulation of Tregs may represent a plausible target for treating ALI.

“Immune conditioning” may be a fashionable concept, but in essence it also points to the intervention of immune cells and their effects. Cell-based therapy for ARDS has the potential to be of therapeutic value (110–113). Various therapeutic approaches with some success in promoting Tregs have been investigated in diseases will provide some important clues to similar treatments for ARDS (114–117). Generally, Tregs have a protective effect and are beneficial to patients with ALI, but excessive suppression of inflammation is not an ideal outcome. Therefore, a dynamic assessment of the immune status in ALI is essential for the elucidation of its Treg-based therapy.

## Author Contributions

FX and SL wrote this manuscript. FX and HW revised this manuscript. FX, CW, and ZX searched and collected bibliography.

## Conflict of Interest Statement

The authors declare that the research was conducted in the absence of any commercial or financial relationships that could be construed as a potential conflict of interest.
